# Development and Genomic Validation of a Population-Specific SNP Panel for Parentage Verification in Chinese Holstein Cattle

**DOI:** 10.3390/ijms27209198

**Published:** 2026-10-16

**Authors:** Jinhuan Dou, Jingyi Gu, Ao Wang, Ziyu Luo, Hetian Huang, Xihui Sheng, Yachun Wang, Ying Yu

**Affiliations:** 1 College of Animal Science and Technology, Beijing University of Agriculture, Beijing 102206, China; doujinhuan@bua.edu.cn (J.D.);; 2 Key Laboratory of Animal Genetics, Breeding and Reproduction, Ministry of Agriculture and Rural Affairs, National Engineering Laboratory of Animal Breeding, College of Animal Science and Technology, China Agricultural University, Beijing 100193, China; lxxkwa@163.com; 3 College of Animal Science and Technology, Henan Agricultural University, Zhengzhou 450046, China

**Keywords:** parentage verification, single nucleotide polymorphism, Chinese Holstein cattle, pedigree accuracy, targeted sequencing, genomic selection

## Abstract

Accurate pedigree information is essential for genetic evaluation and genomic selection in dairy cattle. However, the performance of SNP-based parentage verification depends on marker informativeness, genomic distribution, and compatibility with the target population. In this study, we developed and validated a population-specific 260-SNP panel for parentage verification in Chinese Holstein cattle. The panel was selected from BovineSNP150K genotype data of 2954 Chinese Holsteins based on high marker informativeness, genotyping quality, LD-based control of marker redundancy, and genome-wide distribution. The selected SNPs had a mean minor allele frequency of 0.4981, expected heterozygosity of 0.5000, and polymorphism information content of 0.3750, with a combined probability of exclusion >0.999. Parentage verification of 867 recorded candidate parent–offspring pairs using likelihood-based and Mendelian exclusion-based approaches showed 99.54% agreement, with a Cohen’s κ of 0.981. Random SNP-subset analyses showed greater variation in pedigree concordance estimates with smaller marker sets, whereas larger subsets produced more stable estimates across replicate subsets. Independent validation using targeted-capture sequencing showed a mean SNP detection rate of 99.96% and complete genotype concordance in duplicate tests. Independent validation across six commercial dairy farms identified an overall inconsistency rate of 12.97% between recorded pedigree relationships and genotype-based parentage evidence. These results demonstrate that the population-specific 260-SNP panel provides informative and reproducible genotyping for parentage verification in Chinese Holstein cattle and offers a practical tool for pedigree quality control in dairy cattle breeding programs.

## 1. Introduction

Accurate pedigree information is fundamental to reliable genetic evaluation, breeding value estimation, and genomic selection in dairy cattle. Pedigree inconsistencies can bias estimates of genetic parameters, reduce the accuracy of breeding value prediction, and ultimately compromise genetic gain in breeding populations [1,2,3,4]. With the widespread implementation of genomic information in livestock breeding, pedigree integrity remains important for genetic evaluation, pedigree reconstruction, and the effective integration of genomic and phenotypic information [4,5]. In large-scale dairy production systems, pedigree inconsistencies may arise from errors in artificial insemination records, incomplete farm documentation, sample misidentification, or data transfer, emphasizing the importance of reliable approaches for routine parentage verification.

Parentage verification provides an effective means of identifying pedigree inconsistencies and improving pedigree integrity. Traditional parentage testing in cattle has relied on blood groups and microsatellite markers, but their application to large populations is constrained by labor-intensive procedures, limited scalability, and difficulties in standardization across laboratories [6,7]. With the development of high-throughput genotyping technologies, single nucleotide polymorphisms (SNPs) have become increasingly important for parentage verification because of their genome-wide abundance, low mutation rates, high reproducibility, and compatibility with automated genotyping platforms [8,9,10]. SNP-based data can also support broader pedigree reconstruction and relationship inference when sufficiently informative markers and appropriate statistical approaches are used [10]. International organizations, including the International Society for Animal Genetics (ISAG) and the International Committee for Animal Recording (ICAR), have consequently established standardized SNP sets for cattle parentage verification and discovery [11,12].

The effectiveness of SNP-based parentage verification depends not only on the number of markers but also on their genetic characteristics in the target population. Marker informativeness, allele-frequency distribution, genotyping error, marker density, and the genetic background of the tested population can all influence false-positive and false-negative parentage verifications [13,14]. Previous analyses have shown that panels containing SNPs with relatively high minor allele frequencies generally achieve greater parentage resolution with fewer markers, while the number of SNPs required for robust assignment varies among livestock populations [14,15]. Furthermore, evaluation of East Asian cattle populations has demonstrated that the performance of standardized and population-specific SNP panels can differ according to breed and marker composition [16]. These findings support the use of population-informative SNPs with high polymorphism and broad genomic distribution when designing parentage panels for specific breeding populations.

Chinese Holstein cattle constitute the major dairy population in China, and the increasing implementation of genomic selection further emphasizes the importance of accurate pedigree information. Previous studies in Chinese dairy and cattle populations have demonstrated the feasibility of microsatellite- and SNP-based parentage testing and have identified informative markers for pedigree verification [6,17]. However, many earlier studies involved relatively limited population sizes or lacked independent validation of the selected markers using a separate genotyping technology and commercial dairy populations. In addition, the robustness of parentage verification with respect to marker number has not been systematically evaluated in a large Chinese Holstein dataset. Therefore, the objectives of this study were to develop a population-specific SNP panel for parentage verification in Chinese Holstein cattle using large-scale SNP genotype data; characterize the genomic distribution and genetic informativeness of the selected markers; compare parentage classifications obtained using likelihood-based and Mendelian exclusion-based approaches; evaluate the robustness of parentage-verification results across different SNP subset sizes; and independently validate the genotyping performance and practical applicability of the panel using targeted-capture sequencing in commercial dairy populations.

## 2. Results

### 2.1. Genomic Characteristics of the Population-Specific SNP Panel

**Figure 1:**
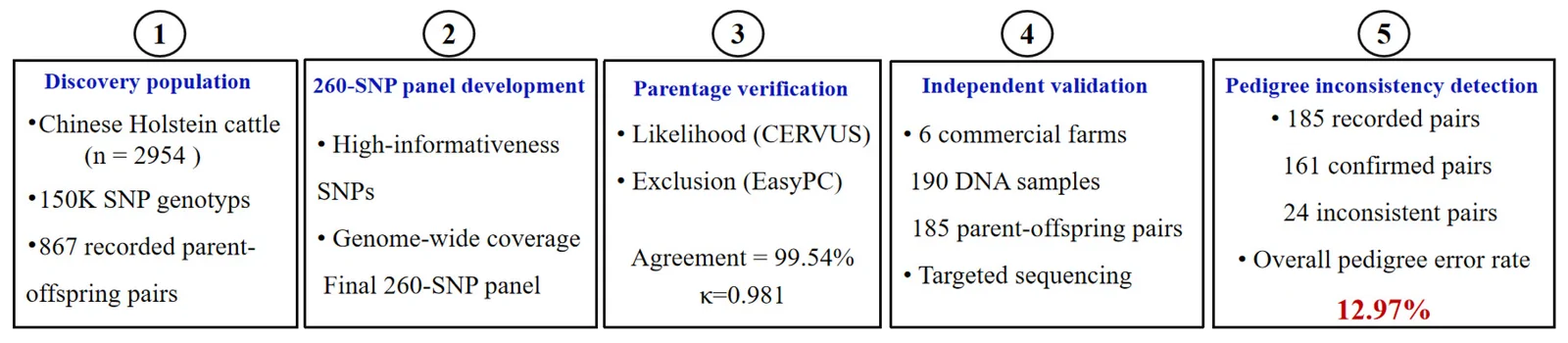
Workflow for development and independent validation of the population-specific SNP panel for parentage verification in Chinese Holstein cattle. The workflow comprised five stages: (1) construction of the discovery population; (2) development of the 260-SNP panel; (3) parentage verification using likelihood-based and Mendelian exclusion-based approaches; (4) independent validation using targeted-capture sequencing in commercial dairy populations; and (5) identification of pedigree inconsistencies.

A population-specific SNP panel was developed from BovineSNP150K genotype data of 2954 Chinese Holstein cattle following sequential filtering for marker informativeness, genotyping quality, LD-based control of marker redundancy, and genome-wide distribution (Figure 1). The final panel comprised 260 SNPs distributed across the 29 bovine autosomes, selected following linkage disequilibrium pruning at a pairwise r^2^ threshold of 0.02 to reduce marker redundancy.

**Figure 2:**
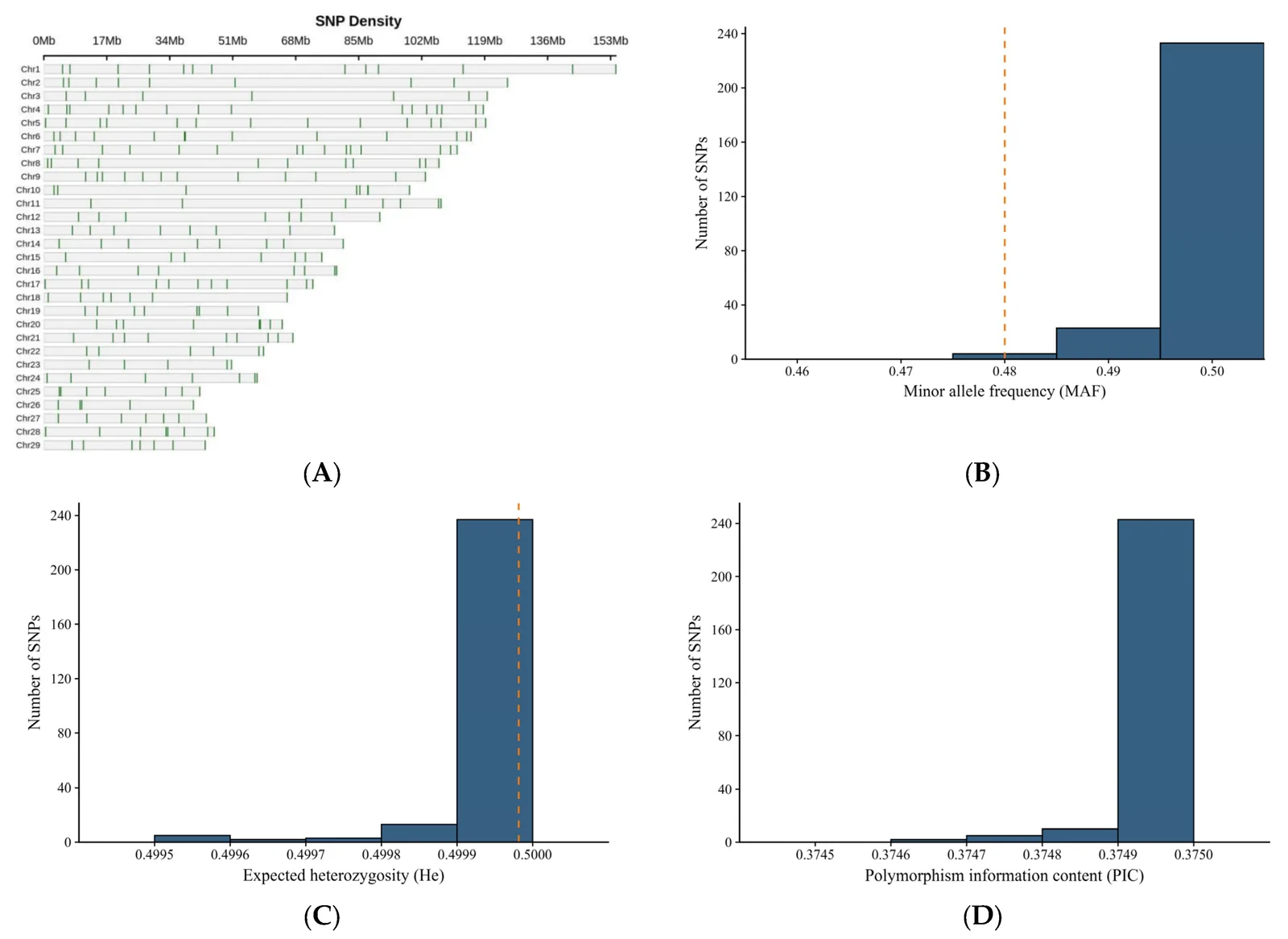
Genomic distribution and population-genetic characteristics of the 260-SNP panel. (**A**) Distribution of the selected SNPs across the 29 Bos taurus autosomes. (**B**) Distribution of minor allele frequency (MAF). (**C**) Distribution of expected heterozygosity (He). (**D**) Distribution of polymorphism information content (PIC).

The selected SNPs showed high levels of genetic informativeness (Figure 2 and Table 1). The mean minor allele frequency (MAF), expected heterozygosity (He), and polymorphism information content (PIC) were 0.4981, 0.5000, and 0.3750, respectively. MAF values ranged from 0.4838 to 0.5000, He from 0.4995 to 0.5000, and PIC from 0.3747 to 0.3750. The CPE is greater than 0.999, indicating an extremely high combined exclusion probability. Only 10 of the 200 SNPs (5.0%) in the parentage verification set and 60 of the 554 SNPs (10.83%) in the parentage discovery set were represented in the genotyping dataset. The limited representation of these reference SNPs precluded a direct performance comparison with the complete ICAR/ISAG panels. Detailed genomic and population-genetic characteristics of the individual SNPs included in the final panel are provided in Table S1.

### 2.2. Parentage Verification Performance and Agreement Between Assignment Methods

**Figure 3:**
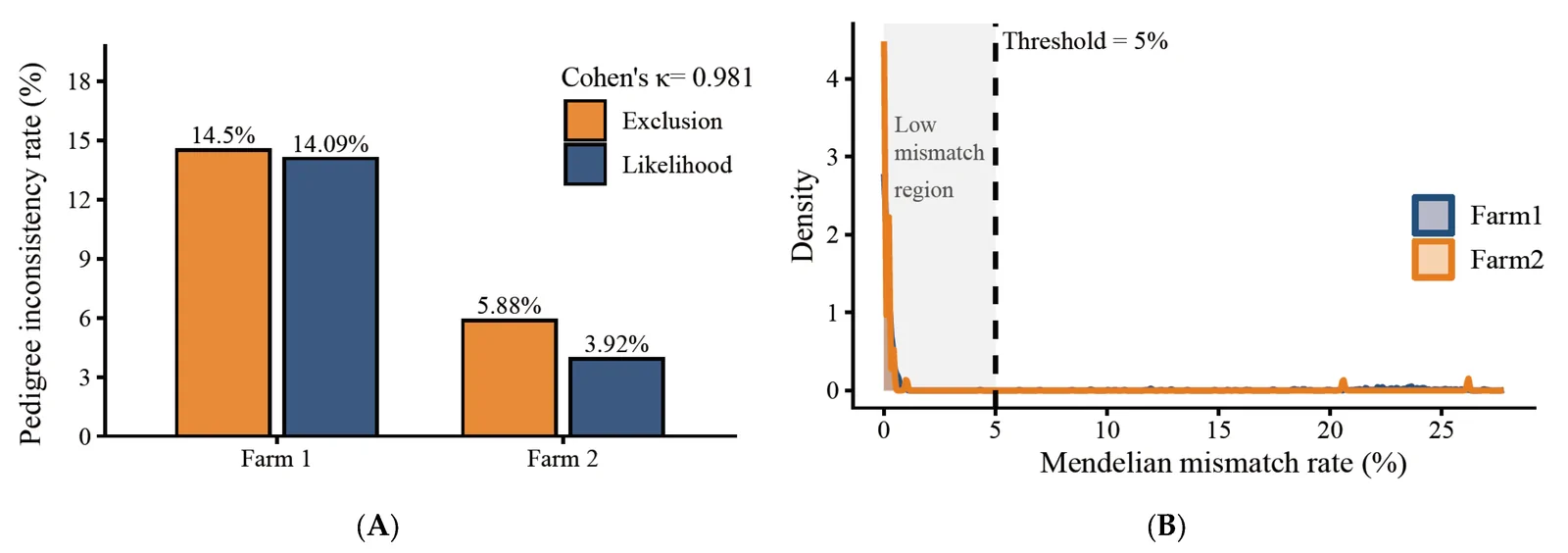
Parentage verification performance in the discovery population. (**A**) Pedigree inconsistency rates obtained using the likelihood-based approach implemented in CERVUS and the Mendelian exclusion-based approach implemented in EasyPC in Farm 1 and Farm 2. (**B**) Distribution of Mendelian mismatch rates among recorded candidate parent–offspring pairs in Farm 1 and Farm 2. The dashed vertical line indicates the 5% mismatch threshold used to classify recorded pairs as concordant (≤5%) or discordant (>5%).

Parentage verification was performed for 867 recorded candidate parent–offspring pairs in the discovery population, comprising 816 pairs from Farm 1 and 51 pairs from Farm 2. The pairs were evaluated independently using the likelihood-based method implemented in CERVUS v3.0.7 and the Mendelian exclusion method implemented in EasyPC v3.6. At the farm level, pedigree inconsistency rates estimated using the two approaches were generally similar, although the exclusion-based approach yielded slightly higher rates than the likelihood-based approach in both farms (Figure 3A). In Farm 1, the pedigree inconsistency rates were 14.50% and 14.09% for the exclusion-based and likelihood-based approaches, respectively, whereas the corresponding rates in Farm 2 were 5.88% and 3.92%. The distributions of Mendelian mismatch rates were strongly concentrated at low values in both farms, whereas a smaller proportion of recorded candidate parent–offspring pairs exhibited mismatch rates above the 5% classification threshold (Figure 3B).

At the individual-pair level, the two approaches showed highly consistent parentage classifications (Table 2). Of the 867 recorded parent–offspring pairs, 746 were classified as concordant and 117 as discordant by both approaches, whereas only four pairs received different classifications. All four discrepancies involved pairs classified as concordant by the likelihood-based approach but discordant by the exclusion-based approach. The overall agreement between the two approaches was 99.54%, with a Cohen’s *κ* of 0.981.

### 2.3. Robustness of Parentage Verification Across SNP Subset Sizes

To assess the robustness of parentage verification with respect to marker number, random subsets containing 25, 50, 100, 150, and 200 SNPs were repeatedly sampled from the complete 260-SNP panel, with 100 replicates generated for each subset size. The complete 260-SNP panel was evaluated without resampling.

**Figure 4:**
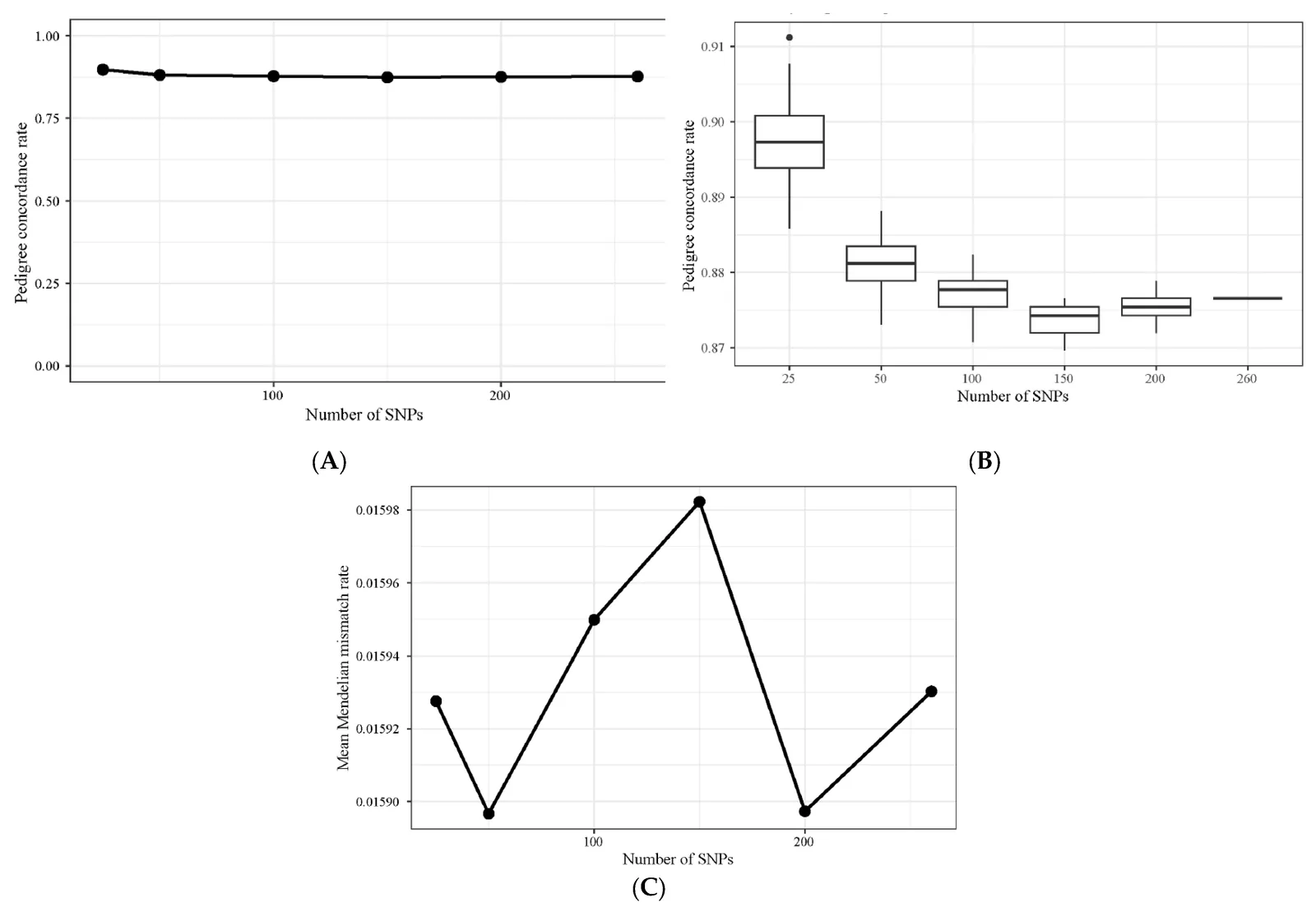
Robustness of parentage verification across SNP subset sizes. (**A**) Mean pedigree concordance across SNP subsets containing 25, 50, 100, 150, or 200 SNPs and the complete 260-SNP panel. (**B**) Distribution of pedigree concordance estimates across 100 randomly generated subsets for each subset size from 25 to 200 SNPs; the complete 260-SNP panel was evaluated without resampling. (**C**) Mean Mendelian mismatch rate across SNP subsets of different sizes. Note: For each subset size from 25 to 200 SNPs, 100 independent subsets were randomly sampled without replacement from the complete 260-SNP panel. The full 260-SNP panel represents a fixed marker set and therefore has no sampling-derived variation. Recorded parent–offspring pairs with Mendelian mismatch rates ≤ 0.05 were classified as concordant.

Pedigree concordance estimates showed greater variation among replicate subsets when fewer SNPs were included, whereas variability generally decreased as marker number increased (Figure 4A,B). Smaller SNP subsets also tended to yield slightly higher pedigree concordance estimates than larger subsets. In contrast, the mean Mendelian mismatch rate remained similar across subset sizes and was approximately 1.59% (Figure 4C). Overall, larger SNP subsets produced less variable concordance estimates, whereas the complete 260-SNP panel was unaffected by random subset-composition variation.

### 2.4. Independent Targeted-Sequencing Validation in Commercial Dairy Populations

The 260-SNP panel was independently evaluated using targeted-capture sequencing in samples collected from six commercial dairy farms located in Beijing, Shanghai, Inner Mongolia, and Xinjiang. Sequencing generated an average of 0.10 Gb of clean bases and approximately 0.68 million reads per sample. The mean Q30 value was 96.68%, and the mean GC content was 45.05%. An average of 689,624 reads per sample were aligned to the reference genome, with an overall mapping rate of 99.9% and a unique mapping rate of 94.29%.

**Figure 5:**
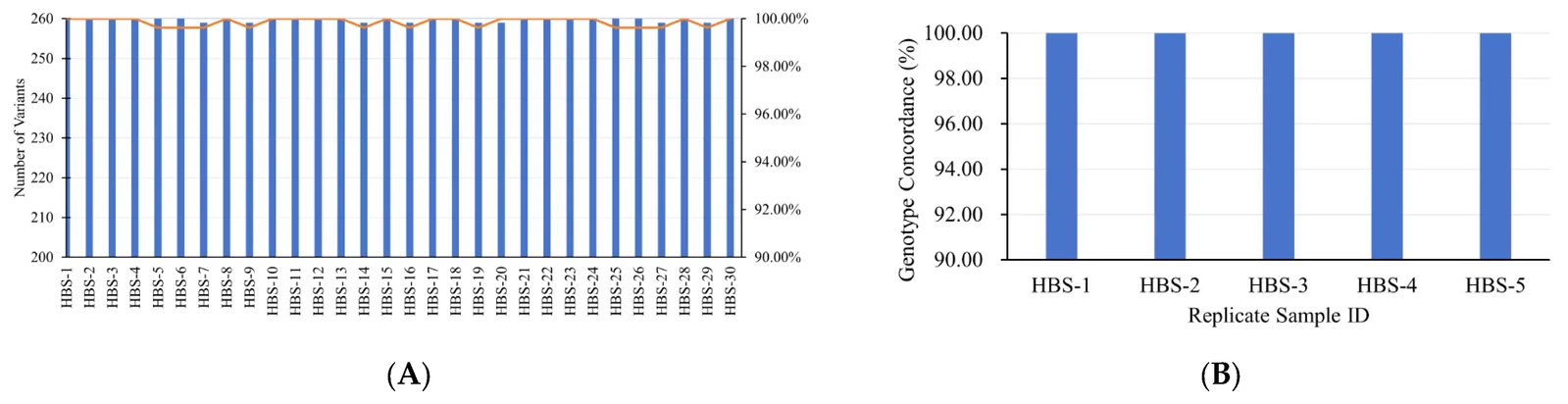
Genotyping performance of the population-specific SNP panel in the independent validation population. (**A**) Number of detected SNPs (blue bars, left y-axis) and SNP detection rates (orange line, right y-axis) in representative targeted-capture sequencing samples. (**B**) Genotype concordance among replicated samples.

Targeted sequencing provided consistently high recovery of the selected loci, with a mean SNP detection rate of 99.96% across validation samples (Figure 5A). Replicate samples showed complete genotype concordance, supporting the technical reproducibility of the sequencing-based genotyping procedure (Figure 5B).

Among the 185 recorded candidate parent–offspring pairs evaluated in the independent validation population, 161 pairs were consistent with the recorded pedigree, whereas 24 pairs showed Mendelian incompatibilities, corresponding to an overall pedigree inconsistency rate of 12.97% (Table 3). Farm-specific inconsistency rates ranged from 4.35% to 19.44%, with the lowest rate observed at the Xinjiang farm and the highest at one of the Beijing farms.

## 3. Discussion

Accurate parentage information remains important for genetic evaluation and genomic breeding, particularly when pedigree records are integrated with genomic data. In the present study, a population-specific SNP panel was developed from large-scale 150 K genotype data of Chinese Holstein cattle and evaluated through several complementary analyses. The selected markers showed high polymorphism and broad autosomal coverage, with a combined probability of exclusion greater than 0.999 (Figure 2; Table 1). Parentage classifications obtained using likelihood-based and Mendelian exclusion-based approaches were highly concordant (Figure 3; Table 2), while the random-subset analysis showed that estimates based on smaller marker sets were more affected by marker composition (Figure 4). Independent targeted-capture sequencing further demonstrated high locus recovery and genotype reproducibility, and application of the panel in six commercial dairy farms identified inconsistencies between recorded pedigrees and genotype-based parentage evidence (Figure 5; Table 3). Taken together, these analyses support the use of the panel as a practical tool for pedigree verification and quality control in Chinese Holstein populations.

The effectiveness of an SNP panel for parentage verification is determined not simply by the number of loci but also by the informativeness and genomic distribution of the selected markers. Allele frequency has a particularly important influence on exclusion power, and SNPs with intermediate allele frequencies generally provide greater discriminatory information for parentage testing [13,14]. Strucken et al. further showed that marker number, allele-frequency distribution, genotyping error, and the allowable mismatch rate jointly affect false-positive and false-negative assignments across livestock populations [14]. In the present panel, deliberate selection for high minor allele frequency, genotyping quality, LD-based control of marker redundancy, and broad genomic distribution resulted in a mean MAF of 0.4981, mean He of 0.5000, mean PIC of 0.3750, and a CPE > 0.999 (Figure 2; Table 1). A similar principle was demonstrated by Hu et al., who developed a 50-SNP parentage panel for a Simmental-Holstein crossbred population using highly informative loci with a mean MAF of 0.43 and achieved high cumulative exclusion probabilities [15]. Population-specific selection may be particularly useful when allele-frequency distributions differ among cattle populations. In Hanwoo and Wagyu cattle, Strucken et al. found that the performance of standardized and population-specific SNP panels depended on both marker composition and panel size, and population-specific markers improved assignment performance when relatively small panels were used [16]. These observations support the view that the performance of the present 260-SNP panel is attributable to the combined informativeness and genomic distribution of its markers rather than to panel size alone.

The close agreement between the likelihood-based and Mendelian exclusion-based analyses provides additional evidence for the consistency of parentage classifications. Likelihood approaches evaluate the relative statistical support for candidate relationships by incorporating allele frequencies and genotyping error, whereas Mendelian exclusion identifies genotype incompatibilities between putative parents and offspring. Despite these methodological differences, the two approaches agreed for 99.54% of the 867 recorded parent–offspring pairs, with a Cohen’s κ of 0.981 (Figure 3; Table 2). The four discrepant classifications indicate, however, that likelihood support and a fixed Mendelian mismatch threshold are not equivalent decision criteria. Such differences are expected because assignment outcomes may also depend on genotype quality, missing data, candidate-parent availability, and the assumptions used by the analytical procedure [9,10]. The marker-number analysis provided a complementary assessment of this robustness. Random subsets containing fewer SNPs showed greater variation in pedigree concordance, whereas variation generally decreased as marker number increased (Figure 4). The slightly higher concordance obtained for some smaller subsets should not be interpreted as improved performance, because a reduced number of loci may simply fail to reveal some Mendelian incompatibilities. Strucken et al. reported the same general dependence of parentage performance on panel size and mismatch criteria and recommended at least 200 SNPs when the aim is to reduce false-negative assignments [14]. Thus, the complete 260-SNP panel used here should be regarded as a fixed, informative marker set that reduces sensitivity to random marker composition, rather than as a statistically defined optimum number of SNPs.

Independent validation was an important part of the present study because marker performance estimated from the same genotyping dataset used for marker discovery does not necessarily establish reproducibility under a different assay system. Targeted-capture sequencing yielded a mean SNP detection rate of 99.96%, and duplicate testing showed complete genotype concordance, indicating reliable recovery of the selected loci on a sequencing platform independent of the original SNP array (Figure 5). More importantly, application of the panel to 185 recorded parent–offspring pairs from six commercial farms identified 24 genetically discordant pairs, corresponding to an overall pedigree inconsistency rate of 12.97% (Table 3). A comparable magnitude of pedigree conflict has been reported in registered Holstein populations. García-Ruiz et al. evaluated 7508 Mexican Holsteins using genomic information and found 777 parent conflicts among 7795 progeny tests, equivalent to a recorded-parentage conflict rate of 9.97% [18]. Hu et al. likewise reported inconsistencies between genetically inferred and recorded parentage in a Simmental-Holstein crossbred population [15]. Such discrepancies are relevant to breeding programs because incorrect pedigree relationships can affect genetic evaluation; recent work has shown that pedigree inconsistencies can influence both genetic evaluations and validation statistics even under single-step genomic evaluation [4]. Nevertheless, the 12.97% observed here should be interpreted as inconsistency between pedigree records and genotype-based evidence rather than as proof of a specific type of recording error. In a recent analysis of 11,224 Australian Angus cattle, Reverter et al. showed that Mendelian inconsistencies can arise from pedigree recording errors, sample mislabeling, or errors in genotypes and genotype imputation [19]. Similar sources may operate in commercial dairy populations, together with animal-identification or data-transfer errors and incomplete specification of candidate parents. The present study was not designed to distinguish among these causes. Accordingly, the principal value of the panel in this context is to identify records requiring further verification before their use in downstream genetic evaluation or genomic breeding analyses.

Several limitations should be considered when applying these results. First, marker selection was based on allele-frequency characteristics and genomic distribution in Chinese Holstein cattle, and equivalent performance should not be assumed for genetically distinct cattle populations. Breed- and population-dependent differences in SNP informativeness and parentage performance have been demonstrated previously [14,16]. Second, only a limited fraction of the ICAR/ISAG reference SNPs was represented in the 150K genotyping dataset used here, preventing a direct head-to-head evaluation of the complete international reference panels and the population-specific panel. The present results therefore do not imply that the ICAR/ISAG panels are unsuitable for Chinese Holstein cattle. Third, the current analysis primarily verified whether recorded parent–offspring relationships were genetically compatible; it did not attempt unrestricted pedigree reconstruction when a recorded parent was excluded. Genomic relationship approaches can extend pedigree validation beyond parent–offspring verification and can identify previously unrecorded relatives when sufficiently dense genotype data and an appropriate reference population are available. Moore et al., for example, used genomic relationships among 5993 Limousin cattle to validate recorded pedigrees and identify previously unrecorded relationships [20], while likelihood-based pedigree reconstruction methods can also accommodate multiple relationship classes and unsampled relatives [10]. Fourth, although the independent validation population included six commercial dairy farms from four regions of China, further evaluation in larger and more geographically diverse Chinese Holstein populations would be valuable for assessing the broader applicability of the panel. Despite these limitations, the combination of population-based marker selection, agreement between two complementary parentage-verification approaches, marker-subset analysis, and validation using a separate sequencing assay provides multiple lines of evidence for the reliability of the panel. Its most immediate application is pedigree quality control in Chinese Holstein breeding populations, where genetically inconsistent records can be flagged for verification before they enter genetic evaluation. This remains relevant in genomic breeding systems because pedigree inconsistencies can continue to affect evaluation and validation when pedigree and genomic information are jointly used [4].

## 4. Materials and Methods

### 4.1. Discovery Population and Genotyping Data

A total of 2954 Chinese Holstein cattle from two commercial dairy farms in Beijing, China, were included in the discovery population. All animals had pedigree records and were genotyped using the Illumina BovineSNP150K BeadChip (Illumina Inc., San Diego, CA, USA). The genotype data from these animals were used for SNP-panel development. Based on the available pedigree and genotype records, 867 recorded candidate parent–offspring pairs were identified, comprising 816 pairs from Farm 1 and 51 pairs from Farm 2. These pairs were subsequently used to evaluate parentage-verification performance, compare assignment methods, and assess robustness across different SNP subset sizes. An independent validation population was subsequently obtained from six commercial dairy farms located in Beijing, Shanghai, Inner Mongolia, and Xinjiang, China. According to farm pedigree records, 185 candidate parent–offspring pairs were available for validation.

### 4.2. SNP Quality Control and Panel Development

Candidate SNPs were selected from the BovineSNP150K genotype dataset according to marker informativeness, genotyping quality, and genome-wide distribution. SNPs were required to have a minor allele frequency (MAF) ≥ 0.45, a polymorphism information content (PIC) ≥ 0.30, and a genotype call rate >0.95. Only autosomal SNPs located on Bos taurus autosomes 1–29 (BTA1–BTA29) were considered.

To reduce local marker redundancy and promote broad genome-wide coverage, SNPs were pruned using a sliding window of 30,000 SNPs, a step size of 1 SNP, and a pairwise r^2^ threshold of 0.02. Marker distribution across the 29 autosomes was also considered to avoid excessive clustering within individual genomic regions. Following these filtering and distribution criteria, 260 SNPs were retained to construct the population-specific parentage-verification panel for Chinese Holstein cattle. Genomic positions of the selected SNPs were based on the Bos taurus UMD3.1 reference genome assembly.

### 4.3. Genetic Diversity and Exclusion Probability

The genetic informativeness of the selected SNPs was evaluated using minor allele frequency (MAF), expected heterozygosity (He), and polymorphism information content (PIC). For a biallelic locus with allele frequencies *p* and *q*, where *p* + *q* = 1, expected heterozygosity was calculated as *He* = 1 − (*p*^2^ + *q*^2^) = 2*pq*. PIC was calculated from the allele-frequency distribution of each locus according to the standard formula for biallelic markers.

For each SNP, the single-parent exclusion probability (PE) was calculated for a candidate parent–offspring comparison when genotype information for the other parent was unavailable, as *PE* = *pq*(1 − *pq*), following Jamieson and Taylor [21]. The combined exclusion probability (CPE) across all loci was calculated as follows:

(1) $$\mathrm{CPE}\;=\;1\;-\prod_{i=1}^{n}\left(1-{\mathrm{PE}}_{i}\right)$$

where *PE_i_* denotes the single-parent exclusion probability of the *i*th SNP and *n* is the total number of loci included in the panel.

### 4.4. Parentage Verification Using Likelihood- and Mendelian Exclusion-Based Approaches

Parentage verification in the discovery population was performed independently using likelihood-based and Mendelian exclusion-based approaches. A total of 867 recorded candidate parent–offspring pairs were included in the comparison. Likelihood-based analysis was conducted using CERVUS v3.0.7 [22]. Allele frequencies were estimated from the discovery population, and the genotyping error rate was set at 0.01. Parentage likelihoods were evaluated using logarithm of the odds (LOD) scores, and the statistical confidence of parentage verifications was determined according to the simulation-based procedure implemented in CERVUS.

Mendelian exclusion analysis was performed using EasyPC v3.6 [23]. For each recorded candidate parent–offspring pair, genotype incompatibilities across the 260 SNP loci were identified according to Mendelian inheritance. The Mendelian mismatch rate was calculated as the proportion of incompatible genotypes among the loci successfully compared between the two individuals. Recorded pairs with a mismatch rate > 0.05 were classified as discordant, whereas those with a mismatch rate ≤ 0.05 were classified as concordant.

Agreement between the two approaches was evaluated by cross-classifying the parentage verifications obtained with CERVUS and EasyPC. Overall agreement was calculated as the proportion of recorded pairs receiving the same classification with both approaches, and Cohen’s κ coefficient was used to quantify agreement beyond that expected by chance [24].

### 4.5. Evaluation of Robustness Across SNP Subset Sizes

To assess the robustness of parentage-verification results to marker number and marker composition, SNP subsets containing 25, 50, 100, 150, and 200 markers were randomly sampled without replacement from the complete 260-SNP panel. For each subset size, 100 independent random subsets were generated. The complete 260-SNP panel was evaluated as a fixed marker set and was therefore not subjected to resampling.

For each random subset, Mendelian mismatch rates were recalculated for the 867 recorded candidate parent–offspring pairs using the same classification criterion as in the complete-panel analysis. Pairs with mismatch rates ≤ 0.05 were classified as concordant, whereas those with mismatch rates > 0.05 were classified as discordant. Pedigree concordance, mean Mendelian mismatch rate, and variation in pedigree concordance among the 100 replicate subsets were summarized for each marker number. This analysis was used to evaluate the sensitivity of parentage-verification results to both SNP number and random marker composition.

### 4.6. Comparison with ICAR/ISAG Reference SNPs

The representation of ICAR/ISAG parentage markers in the genotyping dataset was assessed using the 200-SNP parentage verification set and the 554-SNP parentage discovery set. Marker matching was performed based on SNP identifiers, with marker correspondence cross-checked using available rs identifiers and genomic information to account for differences in SNP nomenclature.

### 4.7. Independent Validation Population and DNA Preparation

Independent validation was conducted using 185 recorded candidate parent–offspring pairs collected from six commercial dairy farms located in Beijing, Shanghai, Inner Mongolia, and Xinjiang, China. To evaluate the technical reproducibility of the targeted-sequencing assay, five of these parent–offspring pairs were selected for duplicate testing.

Genomic DNA was extracted from blood samples using the CW2557Q Blood-Step Mag Bead DNA Kit according to the manufacturer’s instructions. DNA purity was assessed using a NanoDrop spectrophotometer, and DNA concentration was determined using a Qubit dsDNA HS Assay Kit. DNA samples with A260/A280 ratios between 1.8 and 2.0 and concentrations > 10 ng/µL were retained for library preparation and targeted sequencing.

### 4.8. Targeted-Capture Sequencing and SNP Genotyping

Targeted-capture sequencing was used to independently evaluate the genotyping performance of the 260-SNP panel and to verify the recorded parent–offspring relationships in the validation population. Sequencing libraries were prepared using 1 μg of genomic DNA per sample. DNA was fragmented to an average size of approximately 250 bp using the CWseq Universal DirectFast DNA Library Prep Kit, followed by end repair, A-tailing, adapter ligation, and PCR amplification. Biotin-labeled probes targeting the 260 SNP loci were hybridized to the prepared libraries for target enrichment. Following stringent washing to remove nonspecifically bound fragments, captured DNA was eluted and amplified by post-capture PCR. The enriched libraries were subsequently quantified and subjected to quality control.

Sequencing was performed on the DNBSEQ-T7 platform (MGI Tech Co., Ltd., Shenzhen, China) using 150-bp paired-end sequencing. Probe design, library preparation, and sequencing were performed by Compass Biotechnology Co., Ltd. (Beijing, China) [25].

Raw sequencing reads were processed using fastp v0.23.2 [26] to remove adapter-contaminated reads, paired reads in which more than 10% of bases were undetermined (N), and paired reads in which more than 50% of bases had Phred quality scores ≤ 5. Sequencing quality was evaluated using Q20 and Q30 metrics. Clean reads were aligned to the Bos taurus UMD3.1 reference genome (Bos_taurus.UMD3.1.dna.toplevel) using BWA-MEM v0.7.17 [27]. Variant calling was performed using GATK v4.2.0 [28]. SNP genotypes with sequencing depth (DP) ≥ 5, genotype quality (GQ) ≥ 20, and mapping quality (MQ) ≥30 were retained for subsequent validation analyses.

### 4.9. Genotyping Performance and Parentage Verification in the Independent Validation Population

The technical performance of the targeted-sequencing assay was evaluated based on SNP detection rate and genotype concordance between replicate tests. SNP detection rate was calculated as the proportion of the 260 targeted SNP loci for which high-confidence genotypes passing the predefined quality filters were obtained. Technical reproducibility was assessed using the five duplicated parent–offspring tests by comparing genotype calls at successfully genotyped loci between the corresponding primary and duplicate tests.

High-confidence genotypes generated by targeted-capture sequencing were subsequently used to verify the 185 recorded candidate parent–offspring relationships in the independent validation population. Mendelian incompatibilities were evaluated using the same exclusion-based criterion applied to the discovery population. Recorded pairs with Mendelian mismatch rates ≤ 0.05 were classified as genetically concordant, whereas pairs with mismatch rates > 0.05 were classified as discordant. The pedigree inconsistency rate was calculated as the number of discordant recorded candidate parent–offspring pairs divided by the total number of recorded candidate pairs evaluated.

### 4.10. Statistical Analysis

Statistical analyses and data summaries were performed using R v4.4.1 (R Core Team, Vienna, Austria). Descriptive statistics were calculated for marker polymorphism, sequencing performance, parentage-verification outcomes, and SNP-subset analyses. Continuous marker characteristics, including MAF, He, and PIC, were summarized using means and ranges, whereas parentage outcomes were summarized using counts and proportions.

Agreement between likelihood-based and Mendelian exclusion-based parentage classifications was evaluated using the overall agreement proportion and Cohen’s κ coefficient. For the SNP-subset analysis, pedigree concordance and mean Mendelian mismatch rate were calculated separately for each random subset, and variation among the 100 replicate subsets was summarized for each marker number. In the independent validation population, pedigree inconsistency rate was calculated as the number of discordant recorded candidate parent–offspring pairs divided by the total number of recorded candidate pairs evaluated.

## 5. Conclusions

This study developed and independently validated a population-specific 260-SNP panel for parentage verification in Chinese Holstein cattle. The selected markers combined high genetic informativeness with broad autosomal coverage and LD-based control of marker redundancy. Parentage classifications were highly consistent between likelihood-based and Mendelian exclusion-based approaches, and the complete panel showed greater robustness to marker-composition variation than smaller randomly sampled subsets. Independent targeted-capture sequencing further demonstrated high SNP recovery and genotype reproducibility. Application of the panel to six commercial dairy farms identified pedigree inconsistencies in 12.97% of recorded candidate parent–offspring pairs. These findings support the use of the 260-SNP panel as a practical tool for pedigree verification and pedigree data quality control in Chinese Holstein breeding populations. Further validation in broader populations and across additional genotyping platforms would help define its wider applicability.

## 6. Patents

The research reported in this manuscript resulted in a granted Chinese invention patent entitled “Molecular Marker Combination for Parentage Verification in Dairy Cattle and Its Application” (Chinese Invention Patent No. ZL 2025 1 1515889.1; Grant Publication No. CN 121182976 B).

## Figures and Tables

**Table 1:** Population-genetic characteristics of the 260-SNP panel.

Parameter	Value
Number of SNPs	260
Mean MAF	0.4981
MAF range	0.4838–0.5000
Mean He	0.5000
He range	0.4995–0.5000
Mean PIC	0.3750
PIC range	0.3747–0.3750
CPE	>0.999

Note: CPE, combined probability of exclusion; He, expected heterozygosity; MAF, minor allele frequency; PIC, polymorphism information content; SNP, single nucleotide polymorphism.

**Table 2:** Agreement between likelihood-based and Mendelian exclusion-based parentage verifications in the discovery population.

	Exclusion-Based: Concordant	Exclusion-Based: Discordant	Total
Likelihood-based: Concordant	746	4	750
Likelihood-based: Discordant	0	117	117
Total	746	121	867
Overall agreement: 99.54%			
Cohen’s κ: 0.981			

Note: Parentage verifications were evaluated independently using the likelihood-based approach implemented in CERVUS and the Mendelian exclusion-based approach implemented in EasyPC. Concordant indicates that the recorded candidate parent–offspring relationship was supported by the corresponding method, whereas discordant indicates that it was not supported. Agreement between the two approaches was assessed using the overall agreement proportion and Cohen’s *κ* coefficient.

**Table 3:** Parentage verification results in the independent validation populations from six commercial dairy farms.

Region	Farm	Recorded Parent–Offspring Pairs	Genetically Concordant Pairs	Discordant Pairs	Pedigree Inconsistency Rate (%)
Beijing	1	36	29	7	19.44
Beijing	2	20	17	3	15.00
Shanghai	1	26	23	3	11.54
Inner Mongolia	1	30	26	4	13.33
Inner Mongolia	2	50	44	6	12.00
Xinjiang	1	23	22	1	4.35
Total	—	185	161	24	12.97

Note: A recorded candidate parent–offspring pair was classified as discordant when the Mendelian mismatch rate exceeded 0.05. Pedigree inconsistency rate was calculated as the number of discordant pairs divided by the total number of recorded candidate parent–offspring pairs within each farm.

## Data Availability

The data supporting the findings of this study are not publicly available because they contain confidential commercial information provided by the participating dairy farms. Summary data supporting the results are included in the article. Additional data may be available from the corresponding authors upon reasonable request and subject to approval by the participating data providers.

## Supplementary Materials

The following supporting information can be downloaded at: https://www.mdpi.com/article/10.3390/ijms27209198/s1.

## Abbreviations

The following abbreviations are used in this manuscript:

BTA	Bos taurus autosome
CPE	combined probability of exclusion
DP	sequencing depth
GQ	genotype quality
He	expected heterozygosity
ICAR	International Committee for Animal Recording
ISAG	International Society for Animal Genetics
LOD	logarithm of the odds
MAF	minor allele frequency
MQ	mapping quality
PE	probability of exclusion
PIC	polymorphism information content
SNP	single nucleotide polymorphism
